# Impact of Group II Baculovirus IAPs on Virus-Induced Apoptosis in Insect Cells

**DOI:** 10.3390/genes13050750

**Published:** 2022-04-24

**Authors:** Hao Zheng, Yong Pan, Mian Muhammad Awais, Weibin Tian, Jingyang Li, Jingchen Sun

**Affiliations:** Guangdong Provincial Key Laboratory of Agro-Animal Genomics and Molecular Breeding & Subtropical Sericulture and Mulberry Resources Protection and Safety Engineering Research Center, College of Animal Science, South China Agricultural University, Guangzhou 510642, China; zhenghao_scau@163.com (H.Z.); panyongscau@gmail.com (Y.P.); awaismian31@yahoo.com (M.M.A.); tian46102256@163.com (W.T.); skjyli@163.com (J.L.)

**Keywords:** insect cell, group II baculovirus, inhibitor of apoptosis protein, apoptosis

## Abstract

Apoptosis plays an important role in virus-host interactions and is a major element of the insect immune response. Exploring the regulatory mechanisms of virus-induced apoptosis through the expression of apoptotic genes holds important research and application value. Functional research on the reported inhibitor of apoptosis proteins (IAPs) mainly focuses on the group I baculovirus, while the functions of the group II baculovirus IAPs remains unclear. To explore its role in the regulation of the apoptosis of insect cells, we constructed the transient expression vector (pIE1 vectors) and the recombinant baculovirus expressing *Bsiap* genes (from the *Buzura suppressaria* nucleopolyhedrovirus) of the group II baculovirus. Apoptosis gene expression results and the virus-induced apoptosis rate show that the overexpression of BsIAP1 could promote apoptosis in insect cells. However, the overexpression of BsIAP2 and BsIAP3 decreases the expression of apoptotic genes, revealing an inhibitory effect. Results on the impact of baculovirus-induced apoptosis also confirm that BsIAP1 reduces viral nucleocapsid expression and the baculovirus titer, while BsIAP2 and BsIAP3 increase them significantly. Furthermore, compared with single expression, the co-expression of BsIAP2 and BsIAP3 significantly reduces the rate of virus-induced apoptosis and improves the expression of nucleocapsids and the titer of offspring virus, indicating the synergistic effect on BsIAP2 and BsIAP3. In addition, combined expression of all three BsIAPs significantly reduced levels of intracellular apoptosis-related genes (including apoptosis and anti-apoptosis genes), as well as apoptosis rate and progeny virus titer, indicating that life activities in insect cells are also inhibited. These findings reveal the relationship between apoptosis and group II baculovirus IAP, which provide an experimental and theoretical basis for further exploration of the molecular mechanism between group II baculoviruses and insect cells.

## 1. Introduction

Apoptosis is a term employed for programmed cell death, which is a spontaneous and orderly non-inflammatory death of cells that occurs under conditions such as viral infection, DNA damage, hypoxia, and other inducements [1,2]. The apoptosis process involves removing excess metabolites and maintaining homeostasis of the internal environment, and usually occurs during periods of growth, development, and aging [2,3]. The characteristic changes that occur during apoptosis include morphological changes, reduction in cell volume, and formation of apoptotic bodies. In addition, biochemical changes, such as characteristic DNA fragmentation, expression of silenced genes, and synthesis of certain biological macromolecules, are also typical of apoptosis [4]. Further, apoptosis involves a molecular cascade that is strictly controlled by genes and energy-dependent cleavage by proteases [5,6].

IAP’s family (Inhibitor of Apoptosis Proteins, IAPs) is a class of endogenous apoptosis inhibitory proteins, which play a role in preventing apoptosis by inhibiting caspase activity in many species [7]. The novel protein with the function of inhibiting apoptosis was first found in CpGV (*Cydia pomonella* Granulosis Virus) and OpMNPV (*Orgyia pseudotsugata* multinucleocapsid nuclearpolyhedrosisvirus), which were collectively referred to as IAP proteins, and, subsequently, more IAPs were found in BmNPV (*Bombyx mori* nuclearpolyhydrosisvirus) and HycuNPV (*Hyphantria cuea* nucleopolyhedrovirus) [8,9,10]. At present, the variety of IAP proteins with repetitive and conserved sequences have been found in *Caenorhabditis elegans* (*C. elegans)*, *Drosophila*, *Bombyx mori* (*B.mori*), and mammalian cells [11,12]. Baculoviruses encode inhibitors of apoptosis (IAPs), which are classified into five groups, IAP1 to IAP5, based on their amino acid sequences [13,14]. These different types of IAPS share some similar protein domains, including an N-terminal Bir domain and a C-terminal RING finger domain. The Bir domain binds to Zi^2+^ ions and to different receptor proteins, to regulate various anti-apoptotic signaling pathways [15]. The functional study was also shown that IAP3 exhibits strong anti-apoptotic effect in five NPVs including CpGV and OpMNPV [15,16]. However, only IAP1 and IAP2 showed significant anti-apoptotic effects in AnpeNPV (*Antheraea pernyi* nucleopolyhedrovirus) and EPPoMNPV (*Epiphyas postvittana* nucleopolyhedrovirus) [17]. In addition, some studies have shown that IAP2 and IAP3 of LdNPV (*Lymantria dispar* nuclearpolyhedrosisvirus) do not inhibit vAcDp35-induced apoptosis in insect cells, but can induce programmed apoptosis in multiple lepidopteran cell lines when transiently expressed [15]. There are certain reports showing the activity of baculovirus IAPs in regulation apoptosis; more in-depth molecular mechanisms of IAPs and functional studies of IAPs of different species are still lacking.

Since the apoptosis of insect cells, the cascade reaction initiated by the caspase family of proteases is the central link of the apoptosis program. The activation of this program mainly includes the mitochondria-dependent pathway and death receptor-mediated signal transduction pathway, and then activates downstream caspases to regulate cell apoptosis [18]. Therefore, various proteases in the insect caspase family are considered to be the main regulatory genes involved in the apoptosis of insect cells. Among caspase genes, the expression levels of *caspase*-1, *caspase*-2, and *caspase*-9 are often used as markers of cell apoptosis states [19]. *caspase*-1 and *caspase*-2 are downstream of the caspase cascade, and encode major proteases with executive functions in apoptosis, along with *caspase*-9 [15]. The products of the genes *dronc* and *dredd* are similar proteases, and are active factors in apoptotic cells that function to recruit, cleave, and activate precursor caspase proteins [20,21]. Further studies have found that *dronc* and *dredd* genes have extensive homology with the *caspase* gene family, and *dronc* is the first caspase-like steroid regulated to be involved in programmed cell death during insect metabolism [22]. The *dredd* gene is one of the key factors in the apoptosis pathway of insect cell death receptors, also named *caspase*-8, as an initial caspase involved in the apoptosis pathway [23]. The overexpression of the *dredd* gene can significantly promote the apoptosis of drosophila and silkworm cells, which belong to the central effector of the death receptor-mediated apoptosis pathway [24,25]. In general, measuring the expression of apoptotic genes is one of the main strategies to explore the function of IAPs and their regulatory effect on virus-induced apoptosis.

Extensive reports have shown that the function of group I baculovirus IAP is closely related to programmed apoptosis of insect cells. Due to the lack of appropriate research materials and stable, sensitive cell lines, the effect of group II baculovirus IAP on insect cell apoptosis is not clear. In this research, the primary objective is to systematically explore the function of group II baculovirus IAP (BsIAP, inhibitor of the apoptosis protein of *Bs*) and its effect on apoptosis. We firstly overexpressed different BsIAPs (and their co-expressions) by insect cell transient expression vector pIE1 to explore their effects on the molecular expression of the classical apoptosis pathway. Secondly, the stable overexpression of BsIAPs (and their co-expressions) in virus-infected insect cells was achieved by the MultiBac high-efficiency expression system and liposome-free transfection technology. Based on this, we evaluated the impact of overexpression of BsIAPs on the regulation of apoptosis-related genes and the rate of virus-induced apoptosis. In addition, the effect of BsIAPs on the proliferation and replication of baculovirus was further explored by measuring the proliferation rate of progeny nucleocapsid and the expression of VP39 capsid protein. Our results reveal details of the relationship between baculovirus and apoptosis, and provide functional information about group II IAPs.

## 2. Materials and Methods

### 2.1. Bacterial Strains, Plasmids, Viral Bacmid, Genes, Reagents, and Cells

*Escherichia coli* DH10B, BW23474, and Top10 were used to propagate bacmids, R6k-γ, and general plasmids. The dual-purpose pIE1 plasmid is designed for cloning and high-level expression of proteins by transiently transfecting Spodoptera-derived insect cells, which contain an ampicillin resistance gene and a hr5 enhancer upstream of the pIE1 promoter. The plasmids, pFBDM and pUCDM, and modified bacmids with gentamycin or chloramphenicol resistance genes inserted by mini-Tn7 or Cre-loxP transposition were constructed in our previous studies [26,27]. Recombinant bacmids were constructed using the multiple cloning sites of the pFBDM and pUCDM plasmids, to include multiple foreign genes introduced by specific transposition [28,29]. *E. coli* Sw106 containing bacmid, pHelper, and pGB_2_Ωinv was constructed as reported previously; *E. coli* Sw106 can directly infect Sf9 cells and enter the cytoplasm to release recombinant bacmid and generate recombinant baculovirus [28,29]. The genome sequence of the group II baculovirus, BsNPV, has been published (GenBank: KM986882.1), and the viral genome template was provided by Dr. Luo (Guangxi Zhuang Autonomous Region Forestry Research Institute). The genes, *Bsiap1*, *Bsiap2*, and *Bsiap3*, were amplified from the BsNPV genome (see File S1 for the gene sequences). Sequences encoding enhanced green fluorescent protein (*egfp*, GenBank: MH891622.1) and mCherry fluorescent protein (*mCherry*, GenBank: MN052904.1) were amplified using corresponding primers and stored in our laboratory (Table 1), then inserted into plasmids as protein expression markers. Pfu *Taq*, restriction enzymes, and T_4_ DNA ligase were purchased from New England Biolabs (Ipswich, MA, USA), and DL-α-ε Diaminopimelic acid was from Sigma (cat. D1377, St. Louis, USA). Luria Bertani medium (10 g tryptone, 10 g NaCl, 5 g yeast extract in 1 L of broth (pH 7.5)) was used to clone and grow *E. coli* containing plasmids. The *Spodoptera frugiperda*-9 (Sf9; CRL-1711, ATCC) cell line was maintained at 27 ℃ in Grace’s Medium supplemented with 10% fetal bovine serum (FBS; Gibco, Waltham, MA, USA).

### 2.2. Construction of Transient Expression Vector

**Figure 1:**
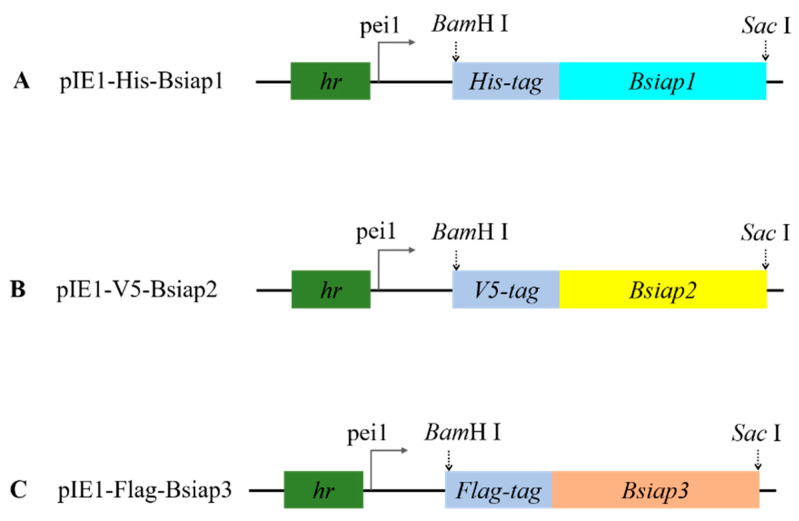
Construction of transient expression vector. Maps of the pIE1-His-Bsiap1 (**A**), pIE1-V5-Bsiap2 (**B**), and pIE1-Flag-Bsiap3 plasmids (**C**).

Transient expression plasmids pIE1-His-Bsiap1, pIE1-V5-Bsiap2, and pIE1-Flag-Bsiap3 were used for the expression of BsIAPs, respectively. Transient expression plasmids were constructed from pIE1, which contains a hr5 enhancer (*hr*) and Multi Cloning Site (MCS) derived from the dual-purpose pIEx/Bac-1 vectors, according to the method described. The gene *Bsiap1* fused with the His-tag was cloned using the primers *Bsiap1*-F and *Bsiap1*-R, shown in Table 1, and introduced into the plasmid pIE1 via the *Bam*H I and *Sac* I restriction sites to generate the recombinant plasmid pIE1-His-Bsiap1 (Figure 1). Similarly, plasmids pIE1-His-Bsiap2 and pIE1-His-Bsiap3 were successfully constructed using the same techniques and restriction sites on the basis of pIE1 vectors (Figure 1).

The three plasmid constructs were transformed into *E. coli* DH5α cells and plasmid DNAs were isolated using the Plasmid Miniprep Kit (Axygen, Corning, NY, USA).

### 2.3. Transfection of Insect Cells

The Sf9 cells were cultured overnight, and the cell suspension was prepared using SF-900 serum-free medium (Thermo Fisher Scientific, Waltham, MA, USA). Monolayer cultures consisting of 10^6^ cells were prepared in petri dishes with a bottom area of 35 mm. The successfully constructed transient expression plasmids were mixed with Lipofectamine 3000 Reagent (Invitrogen, Carlsbad, CA, USA) and transfected into Sf9 cells as required.

### 2.4. Quantitative PCR

RNA reverse transcription and quantitative real-time PCR were conducted to determine the expression levels of *caspase, dronc, dredd,* and *vp39* in the transfected Sf9 cells (concentration 10^6^ cells/mL). Supernatants and cell pellets were separated by centrifugation at 4 ℃, 1500× *g*, 48 h post-transfection. Supernatants were collected for subsequent titer determination, and RNA extracted from cell pellets using Trizol reagent and reverse transcribed with Oligo dT primers (Perfect Real Time, Takara, Shiga, Japan). The resulting cDNA samples were used as a template for quantitative polymerase chain reaction (Q-PCR), using SYBR Premix Ex Taq^TM^ (Tli RNaseH Plus, Takara, Shiga, Japan) and specific primers (Table 2). The control group was transfected with pIE1 plasmid, and the same operation as above was done. Meanwhile, we used α-Tubulin as an internal gene to compare the differences in gene expression.

### 2.5. Construction of Baculovirus Donor Vectors Bearing Bsiaps Expression Cassettes

**Figure 2:**
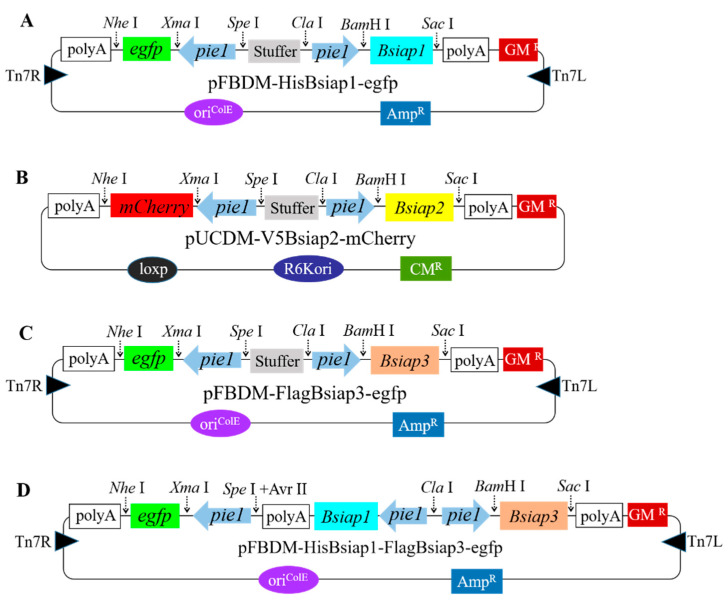
Construction of recombinant plasmids carrying *Bsiaps* and fluorescent genes. Maps of the pFBDM-HisBsiap1-egfp (**A**), pUCDM-V5Bsiap2-mCherry (**B**), pFBDM-FlagBsiap3-egfp (**C**), and pFBDM-HisBsiap1-FlagBsiap3-egfp plasmids (**D**).

To further explore the effect of BsIAPs on baculovirus-induced apoptosis, the donor plasmids were used to construct the recombinant bacmids expressing *Bsiap* genes under the control of the early promoter *pie1*. The donor plasmid, pFBDM-HisBsiap1-egfp (Figure 2A), was used to construct a recombinant baculovirus, BV-HisBsiap1, to explore the effect of BsIAP1 on Sf9 cell apoptosis. The N-terminus of *Bsiap1* was fused with a His tag (amplified with the primers, *Bsiap1*-F and *Bsiap1*-R, Table 1) and cloned into the *Bam*H I and *Sac* I sites of the pFBDM plasmid, under the control of the *pie1* promoter. Further, *egfp* (amplified with primers, *egfp*-F and *egfp*-R, Table 1) was ligated downstream of the *pie1* promoter in the *Xma* I and *Nhe* I sites. The primers *Bsiap2*-F and *Bsiap2*-R were used to amplify *Bsiap2*, fused with a V5 tag, to construct the donor vector pUCDM-V5Bsiap2-mCherry (Figure 2B). *Bsiap2* was ligated into the *Bam*H I and *Sac* I sites, and the *mCherry* expression cassette (amplified with primers, *mCherry*-F and *mCherry*-R, Table 1) was ligated downstream of *pie1* in the *Xma* I and *Nhe* I sites. The His*Bsiap1* gene was replaced by Flag*Bsiap3*, using the restriction sites *Bam*H I and *Sac* I, to construct the recombinant donor plasmid, pFBDM-FlagBsiap3-egfp (Figure 2C). The donor vector, pFBDM-HisBsiap1-egfp, was digested with *Cla* I and *Avr* II to release the DNA fragment, pie1-HisBsiap1-polyA, which was then cloned into the *Cla* I and *Spe* I sites of pFBDM-FlagBsiap3-egfp to construct the donor vector, pFBDM-HisBsiap1-FlagBsiap3-egfp (Figure 2D).

### 2.6. Introduction of Bsiaps into a Bacmid

**Figure 3:**
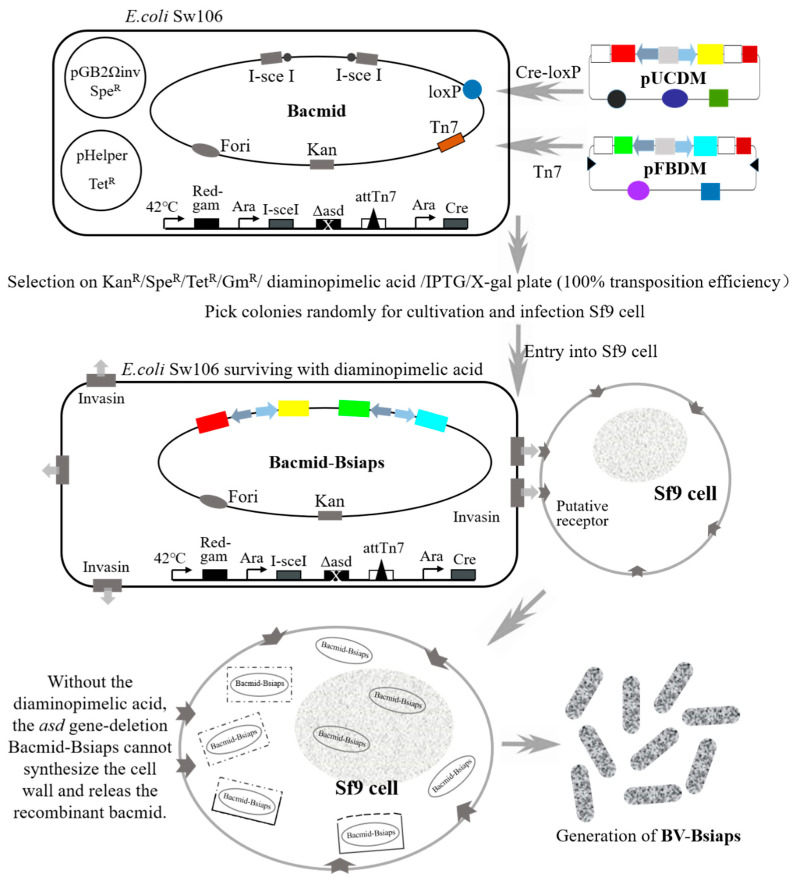
Schematic illustrating the construction of recombinant BV-Bsiaps baculovirus through multi-gene co-expression and liposome-free transfection technology. *Bsiap* genes carried by the recombinant plasmids, pFBDM and pUCDM, were introduced into an *E. coli* Sw106 bacmid using Tn7 and Cre-loxP sites, respectively. Due to the lack of diaminopimelic acid in Sf9 cells, *E. coli* Sw106, which invade Sf9 cells via invasin activity, cannot synthesize cell walls, and bacmids are released and BV-Bsiaps proliferation completed.

Gene cassettes for expression of *Bsiap1*, *Bsiap2*, and *Bsiap3* were introduced into an *E. coli* Sw106 bacmid by mini-Tn7 and Cre-loxP transposition, as described previously [27,28,29]. Recombinant colonies were grown in agarose solid culture medium containing antibiotics, including gentamicin, chloramphenicol, kanamycin, tetracycline, spectinomycin, and ampicillin (Figure 3). Positive clones were screened and identified through blue-white colony selection and specific gene PCR amplification, to generate four recombinant bacmids: bacmid-Bsiap1, bacmid-Bsiap2, bacmid-Bsiap3, and bacmid-Bsiap1-Bsiap3. Bsiap2 was introduced into *asd*-deleted *E. coli* Sw106 (containing bacmid-Bsiap1 introduced using mini-tn7) by Cre-loxP transposition to construct recombinant bacmid-Bsiap1-Bsiap2; bacmid-Bsiap2-Bsiap3 and bacmid-Bsiap1-Bsiap2-Bsiap3 were constructed and screened using similar methods.

### 2.7. Production of Recombinant Baculovirus

*E. coli* Sw106 containing recombinant bacmids with *Bsiap* gene expression cassettes (*Bsiap1*, *Bsiap2*, *Bsiap3*, *Bsiap1*+*Bsiap2*, *Bsiap1*+*Bsiap3*, *Bsiap2*+*Bsiap3*, and *Bsiap1*+*Bsiap2*+*Bsiap3*) were grown until the culture had OD_600_ values of 0.5–1. Bacteria were then collected by centrifugation (3000× *g*) and resuspended in serum-free Grace’s insect medium. Bacterial suspensions were adjusted to different densities (10^4^–10^8^ cell/mL) with serum-free Grace’s insect medium [30]. Exponential growth phase Sf9 cells were cultured overnight in 12-well plates (Thermo Fisher Scientific, Waltham, MA, USA) until cell density reached 70–80%. Different concentrations of bacteria were incubated with Sf9 cells at 28 ℃ for 4–5 h to allow them to invade cells. Bacteria in each well were washed with serum-free Grace’s insect medium and 500 μL fresh Grace’s insect medium (containing 10% FBS and 0.075% penicillin) was added, followed by incubation for 2–4 days. GFP (Green Fluorescent Protein) and mCherry (mCherry Fluorescent Protein) expression were observed using an inverted fluorescence microscope (Eclipse Ti-S, Nikon, Tokyo, Japan) to determine whether recombinant baculoviruses were successfully constructed and infected into Sf9 cells. Recombinant baculovirus were collected from supernatants following centrifugation, and used to re-infect normal Sf9 cells, in order to observe intracellular fluorescent proteins. The above-mentioned procedure was repeated 2–3 times until expression of fluorescent protein was stable, and then supernatants were collected to obtain recombinant baculovirus (BV) with high infectivity (Figure 3).

### 2.8. Western Blot Analysis

Purified baculoviruses and infected cell lysates were subjected to 10% sodium dodecyl sulfate-polyacrylamide gel electrophoresis and transferred to nitrocellulose membranes. Primary antibodies (1:4000 dilution), including against 6×His (Beyotime, Shanghai, China), V5 (Thermo Fisher, Waltham, MA, USA), and Flag (Beyotime, Shanghai, China) tags, were used to detect fusion proteins by western blot analysis. The secondary antibodies were goat anti-pig and goat anti-mouse IgG conjugated to HRP (1:3000 dilution, Thermo Fisher, Waltham, MA, USA). Protein bands were visualized using the ECL chemiluminescence system and Hyper-Max films, as recommended by the manufacturer.

### 2.9. Determination of Recombinant Baculovirus Titer

Sf9 cells in an early exponential phase of growth were diluted to 10^6^ cells/mL with Sf-900 II serum-free medium (SFM, Thermo Fisher, Waltham, MA, USA), and then 100 µL aliquots was placed into microwells of 96-well plates. The genomes of baculoviruses released from cell culture media were collected using a viral genome extraction kit, and then quantitatively detected using baculovirus-specific primers (Table 2, *baculovirus*-F and *baculovirus*-R). Serial baculoviral 10-fold dilutions (to 10^−8^) were prepared in SFM. Once cells were attached to plates, the medium supernatant was removed and 100 µL of each virus dilution was added to the cell monolayers (12-wells per dilution). Following incubation at 28 °C for 4 h, supernatants in each well were replaced with 100 µL fresh insect medium. Plates were checked daily for 2–4 days until fluorescence levels reached a maximum. Baculovirus titers are expressed as 50% tissue culture infective dose (TCID_50_), according to the standard method of Reed and Muench [31].

### 2.10. Determination of Sf9 Apoptosis Rate by Flow Cytometry

The early apoptosis rate of Sf9 cells was determined by flow cytometry analysis, using an Annexin V-PE/7-AAD detection kit (Sino Biological, Wayne, PA, USA). Since Annexin V-PE has a high affinity for phosphatidylserine, it can bind to the surface of Sf9 cell membranes during the early stage of apoptosis. By simultaneously excluding cells containing the nuclear dye 7-ADD with a narrower emission spectrum, apoptotic cells and necrotic cells can be distinguished. Fluorescence in cells (concentration 10^6^ cells/mL) was observed using a fluorescence microscope after infection for 48 h (multiplicity of infection (MOI) = 1), indicating that cell infection and recombinant baculovirus gene expression were successfully achieved. Cells were washed three times with PBS, and centrifuged at 1000× *g* for 5 min at 4 °C for collection as pellets. First, cells were resuspended in 250 μL of Annexin V-PE binding buffer (1:3 dilution) and adjusted to a concentration of 10^6^ cells/mL. Second, 100 μL of the cell suspension was transferred into a 5 mL round-bottom tube (Coring Falcon, Corning, NY, USA), and 5 μL Annexin V-PE and 10 μL 7-AAD solutions added successively, followed by incubation in the dark for 15–20 min. Finally, 400 μL of PBS solution was added, and the apoptosis rate was immediately analyzed by flow cytometry. To exclude the effects of AcMNPV genes on Sf9 cell apoptosis, untreated Sf9 cells were infected with the same titer of wild-type baculovirus AcMNPV-egfp as in the control group. The construction of baculoviruses AcMNPV-egfp and AcMNPV-mCherry is described in our previous publications [30,32]. In addition, untreated Sf9 cells and Sf9 cells that had been infected with wild-type baculovirus were analyzed to assess compensatory regulation.

### 2.11. Statistical Analysis

All values are expressed as mean and standard deviation (SD). GraphPad Prism 7 software was used for data analysis. Significant differences were determined by one-way ANOVA or Kruskal–Wallis’s test. *p* < 0.05 and *p* < 0.01 were considered statistically significant.

## 3. Results

### 3.1. Transfection and Identification of BsIAPs

**Figure 4:**
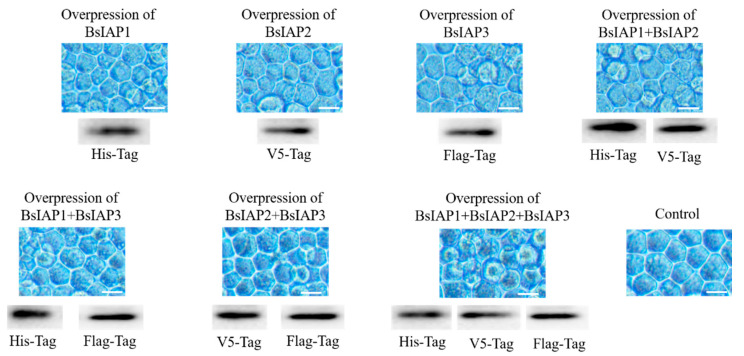
Transfection of insect Sf9 cells and identification of BsIAPs by western blot. The cells of the control group were cultured for the same time after transfection with pIE vector. After the transient expression plasmids were transfected into Sf9 cells (48 h, Bar = 25 μm), the intracellular BsIAPs were identified by western blot with three anti-tag antibodies (His, V5, and Flag), respectively. Western blot identification showed that different BsIAPs (and their combinations) were highly expressed in insect Sf9 cells after co-transfection (see the Figure S1 for details).

Three transient expression plasmids were co-transfected with Sf9 cells to obtain multiple groups of Sf9 cells overexpressing BsIAP1, BsIAP2, BsIAP3, BsIAP1+BsIAP2, BsIAP1+BsIAP3, BsIAP2+BsIAP3, BsIAP1+BsIAP2+BsIAP3 proteins, respectively. Western blot with three anti-tag antibodies (His, V5, and Flag) showed that BsIAPs were successfully expressed with high amounts in cells after transfection (Figure 4). In addition, in order to proofread the effect of the overexpression vector on the regulation of apoptosis genes in Sf9 cells, the control group directly transfected pIE1 into Sf9 cells, and performed the same operation as above.

### 3.2. Influence of BsIAPs on Intracellular Apoptosis Gene Expression

**Figure 5:**
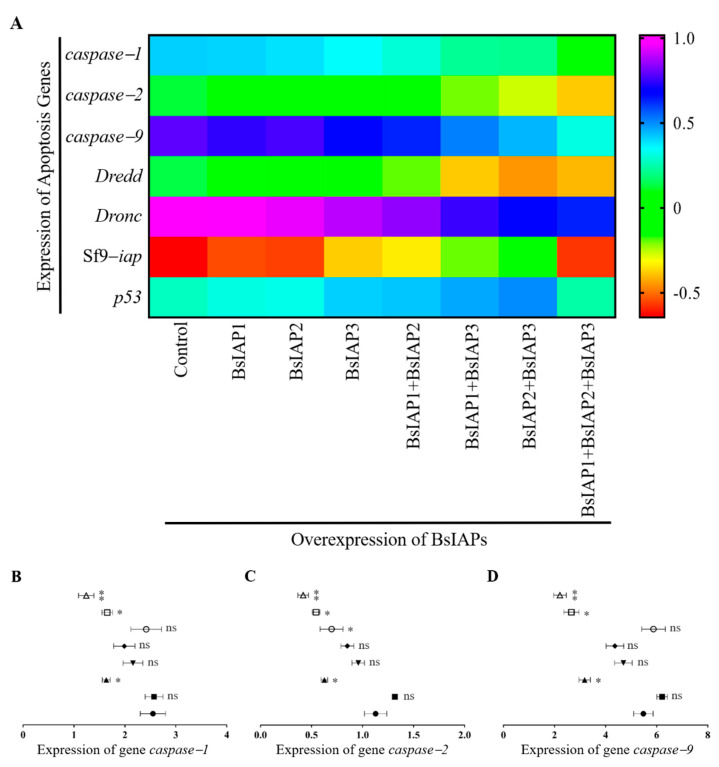

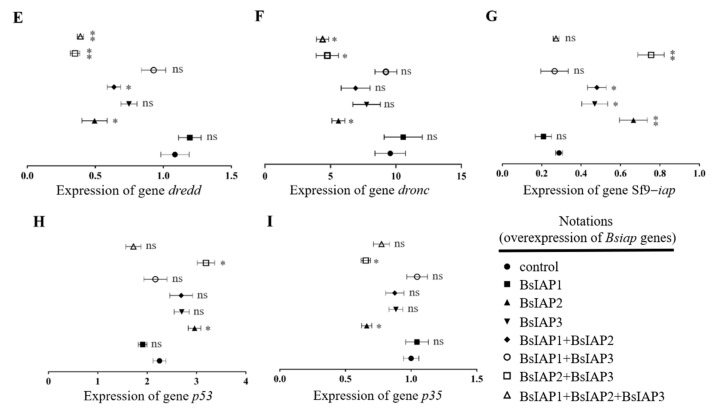
Impact of transient overexpression of BsIAPs on apoptotic gene expression. The expression of apoptosis genes, including the control group, were relative to those of non-transfected insect Sf9 cells. (**A**) Heatmap illustrating the effect of transient overexpressing BsIAPs on apoptotic gene expression. (**B**–**I**) Detailed results of the effects of transient overexpressing BsIAPs on the expression of *caspase*−1 (**B**), *caspase*−2 (**C**), *caspase*−9 (**D**), *dredd* (**E**), *dronc* (**F**), Sf9−*iap* (**G**), and *p53* (**H**). * *p* < 0.05, ** *p* < 0.01.

To explore the regulation of apoptosis by transient overexpression of BsIAPs, the expression of apoptosis genes was determined by Q-PCR in both transfected and non-transfected cells (Table 2). Compared with controls (Figure 5A), overexpression of BsIAP1 increased the expression of the apoptotic genes, *caspase*, *dreed*, and *dronc*, to varying degrees, and decreased the expression of the anti-apoptotic genes, *p53* and *iap*, in Sf9 cells (Sf9-*iap*), indicating that BsIAP1 may promote apoptosis. By contrast, overexpression of BsIAP2 and BsIAP3 significantly reduced the expression levels of *caspase*, *dreed*, and *dron*, and significantly increased those of Sf9-*iap* and *p53*, suggesting that they may strongly inhibit apoptosis (*p* < 0.05). In addition, given the clear differences between BsIAP2 and BsIAP3 in regulation of *caspase*, *dreed*, and *dronc* gene expression, the results show that the apoptosis inhibition effect of BsIAP2 is stronger than that of BsIAP3. Further experiments demonstrated that co-expression of BsIAP2 and BsIAP3 led to significantly stronger inhibition of apoptosis genes than each construct individually, indicating a clear synergistic interaction between these two BsIAPs. Interestingly, co-transfection with simultaneous expression of BsIAP1, BsIAP2, and BsIAP3 led to significantly reduced apoptosis-related (including pro- and anti-apoptotic) gene expression. We speculate that this may be due to disordered intracellular gene expression, caused by joint regulation of multiple genes (see Figure 5B–I for details).

### 3.3. Generation of Recombinant Baculovirus for Stable Expression of BsIAPs

**Figure 6:**
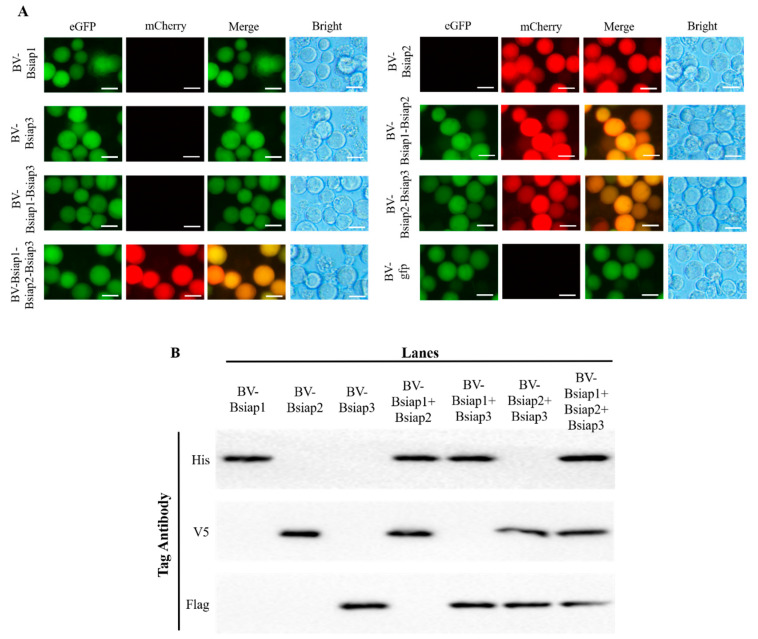
Construction of recombinant baculovirus and identification of BsIAPs. (**A**) Expression of eGFP and mCherry were observed by fluorescence microscopy, indicating that the seven recombinant baculoviruses successfully infected Sf9 cells (Bar, 25 μm). Control cells were infected with BV-gfp under the same operation. (**B**) Western blot analysis using the three anti-tag antibodies (His, V5, and Flag) showed that BsIAPs were highly expressed after 72 h of Sf9 cell infection with the seven recombinant baculoviruses. The abscissa shows the cell samples infected by the seven groups of baculoviruses (BV) respectively. The ordinate indicates that different samples were identified by western blot using three tag antibodies. The result shows that different BsIAP proteins (or their combinations) are successfully and efficiently expressed in cells (**B**).

Recombinant bacmids containing *Bsiaps* expression cassettes were successfully constructed and directly transfected into cultured Sf9 cells via the invasin protein. In the absence of diaminopimelic acid, both in the cell culture medium and intracellularly, *asd*-deficient *E. coli* cannot synthesize a cell wall; therefore, they become disrupted and release the recombinant bacmid, which generates infective recombinant baculovirus particles in Sf9 cells (Figure 6).

Green fluorescence was detected in infected Sf9 cells (measured at 488 nm; red measured at 512 nm), indicating that the recombinant baculovirus carrying *Bsiaps* had successfully invaded the Sf9 cells (Figure 6A). Control cells were infected with recombinant baculovirus expressing only green fluorescent protein (BV-gfp) under the same conditions. When fluorescence reached a maximum, culture supernatants were collected and centrifuged at 80,000× *g*, and mature baculoviral particles were successfully obtained in the pellets. Purified recombinant bacmids were transfected into Sf9 cells to produce seven types of recombinant baculovirus as follows: BV-Bsiap1, BV-Bsiap2, BV-Bsiap3, BV-Bsiap1-Bsiap2, BV-Bsiap1-Bsiap3, BV-Bsiap2-Bsiap3, and BV-Bsiap1-Bsiap2-Bsiap3. Recombinant baculoviruses were collected in the cell supernatants and Sf9 cells were re-infected for 72 h (MOI = 1). Cell pellets were analyzed by western blot, using antibodies against the His, V5, and Flag protein tags, as appropriate, and the results showed that the seven recombinant baculoviruses successfully expressed the target proteins BsIAP1, BsIAP2, and BsIAP3 (Figure 6B).

### 3.4. Influence of BsIAPs on Virus-Induced Apoptosis Rate of Sf9 Cells

**Figure 7:**
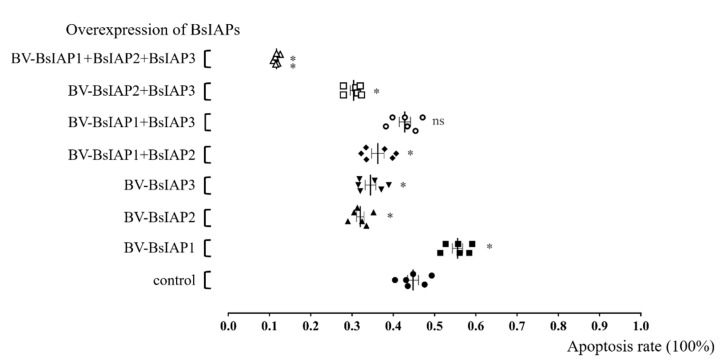
Effects of overexpression of BsIAPs on Sf9 cell apoptosis rate. * *p* < 0.05, ** *p* < 0.01. After Sf9 cells were infected with recombinant baculovirus stably expressing BsIAPs for 48 h, the apoptosis rate was determined by flow cytometry. Control cells were infected with BV-gfp under the same operation.

To further explore the effect of BsIAPs on virus-induced apoptosis rate, flow cytometry and an apoptosis detection kit were used to complete the detection (see the Figure S2 for details). At the same time, we used normal cells and baculovirus (BV-egfp)-infected cells (10^6^ cells/mL, MOI = 1) as the control group, excluding the interference of wild-type baculovirus-induced apoptosis and natural apoptosis. The results showed that overexpression of BsIAP1 significantly promoted the apoptosis of Sf9 cells induced by the baculovirus, which was 28.96% higher than that of controls (Figure 7). Furthermore, expression of BsIAP2 or BsIAP3, individually, significantly reduced the virus-induced Sf9 apoptosis rate by 28.09% and 10.62%, respectively, while co-expression of BsIAP2 and BsIAP3 had a significantly greater effect than that of each molecule singly, with an apoptosis rate 28.89% lower than controls. In addition, the recombinant baculovirus, expressing three different BsIAPs-infected Sf9 cells, significantly inhibited the virus-induced apoptosis of Sf9 cells, leading to rates 72.4% lower than those of controls (*p* < 0.01, Figure 7). Combined with the above-mentioned Q-PCR transient expression results, these data suggest that the combined expression of *Bsiaps* genes caused disordered gene expression, delaying apoptosis, and other intracellular processes.

### 3.5. Effect of BsIAPs on Baculovirus Proliferation Efficiency

**Figure 8:**
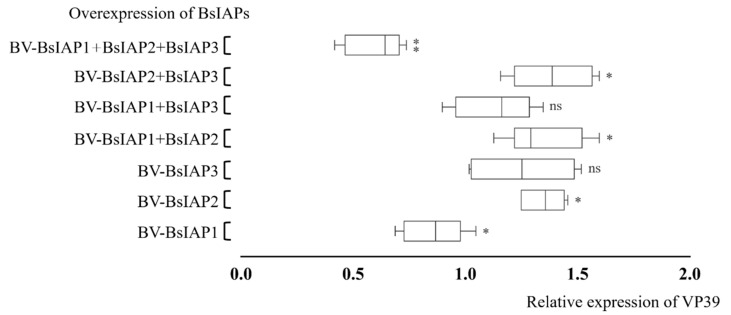
Effects of BsIAP overexpression on VP39 capsid protein levels. * *p* < 0.05, ** *p* < 0.01. After 48 h of infection with recombinant baculovirus stably expressing BsIAPs, total RNA was extracted, and the expression of *vp39* gene was determined by reverse transcription PCR. The VP39 expression is relative to the control group (baculovirus AcBV-gfp expressing fluorescent protein only).

VP39 protein is an important insect baculovirus capsid structural protein that exhibits a high degree of conservation, and can be used to determine the proliferation rate of progeny nucleocapsid [33,34]. Q-PCR analysis 48 h after virus infection showed that overexpression of BsIAP1 significantly reduced VP39 levels, while overexpression of BsIAP2 or BsIAP3 increased VP39 expression (Figure 8). Further, co-expression of BsIAP2 and BsIAP3 significantly increased VP39 expression, indicating that this combination can significantly inhibit Sf9 cell apoptosis and prolong VP39 intracellular replication time. In addition, co-expression of the three BsIAPs disrupted normal gene expression in Sf9 cells, resulting in extremely low VP39 levels (*p* < 0.01).

**Figure 9:**
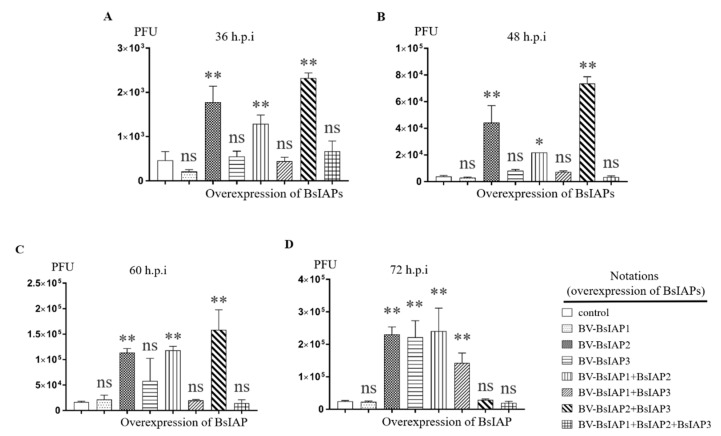
Effects of overexpression of BsIAPs on progeny virus titers after different infection time periods (* *p* < 0.05, ** *p* < 0.01). (**A**) Overexpression of BsIAPs for 36 h. (**B**) Overexpression of BsIAPs for 48 h. (**C**) Overexpression of BsIAPs for 60 h. (**D**) Overexpression of BsIAPs for 72 h. Control cells were infected with BV-gfp under the same operation.

To more clearly reveal the effects of different BsIAPs on virus-induced apoptotic cells, TCID_50_ was used to determine nucleocapsid proliferation rates at different times. Referring to Section 2.9, the baculovirus released from the supernatant of cells expressing BsIAP was collected, and the baculovirus genome in the cell supernatant was quantified using baculovirus-specific primers (Table 2), and the virus was subsequently adjusted to the same amount (10^4^ PFU). The TCID_50_ results showed that virus titer of offspring was lower 36–48 h after overexpression of BsIAP1 than that in controls (Figure 9A,B); however, the difference decreased gradually within 60–72 h after infection, indicating that the mechanism by which BsIAP1 promotes apoptosis may exert its influence at an early stage (Figure 9C,D). In contrast, baculovirus expressing BsIAP2 and BsIAP3 had stably and significantly higher titers than controls at 36–72 h after infection of Sf9 cells (Figure 9). We speculate that the main reason for this finding is that overexpression of BsIAPs leads to alteration of the apoptosis rate, which affects nucleocapsid protein replication and assembly, resulting in altered progeny virus titer during different time periods. Further experiments showed that combined overexpression of BsIAP2 and BsIAP3 resulted in a higher nucleocapsid replication rate, which significantly surpassed that induced by individual BsIAP expression (Figure 9). These results verify our findings from Q-PCR and flow cytometry analysis of apoptotic genes. In addition, combined overexpression of BsIAP1+BsIAP2+BsIAP3 also significantly reduced nucleocapsid protein expression and virus titer, indicating that the expression of intracellular genes was disordered and that normal insect cell physiological processes were delayed.

## 4. Discussion

As an important part of insect immune responses, apoptosis can effectively resist baculovirus proliferation and replication following the invasion. The co-existence of baculovirus and apoptosis (or anti-apoptosis) genes suggests that there may be a complex interacting relationship, providing a potential research entry point for the study of apoptosis. In this study, the transient expression vector pIE1 and MultiBac co-expression technology were used to express various group II baculovirus iap genes in insect Sf9 cells. These two efficient and rapid expression methods provided a stable experimental platform for research into virus-induced insect cell apoptosis.

**Figure 10:**
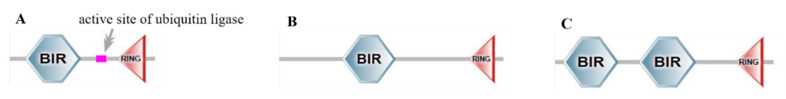
Analysis of BsIAPs amino acid domains and active sites. (**A**) BsIAP1. (**B**) BsIAP2. (**C**) BsIAP3.

In this study, it was also found that the transient expression of BsIAP1 could increase the expression of *caspase* and *dredd*, and induce apoptosis of Sf9 cells. This phenomenon was confirmed by the results of the overexpression of IAP1 of AcMNPV, BmNPV, Hycunpv, and Opmnpv, which could induce apoptosis of Sf9 cells [14]. Similarly, the results that type II baculovirus IAP1 protein stimulates the activity of caspase-3 protease also exist in cell lines such as the *B.mori*, *L. dispar* and *Spilosoma imparis* cell lines [35,36]. The active site analysis of BsIAP1 protein showed that there was an active site of ubiquitin ligase between the Bir and Ring domains of BsIAP1 (Figure 10). Since ubiquitinated protease can affect cell cycle processes such as apoptosis and phagocytosis through transcription, signal transduction, and protein modification, we speculate that the active site of ubiquitinated protease may be one of the active regions promoting apoptosis of BsIAP1 [37,38,39,40]. Based on the fact that group II baculovirus can assist in apoptosis by expressing cofactors (such as Cp94 protein), the transient expression of BsIAP1 cannot significantly promote the apoptosis of Sf9 cells [41].

BsIAP2 and BsIAP3 proteins contain two classes of motifs: a ca. 70 amino acid motif known as a baculoviral IAP repeat Bir at the N-terminal region and a C-terminal Ring domain (Figure 10). Both are required for functionality, but not all RING domains of IAPs are needed for anti-apoptotic function [42,43]. Functionally, the Ring domain is suggested to be involved in the E2 ubiquitin-conjugating function, E3 ubiquitin ligase activities, and the transfer of ubiquitin to target proteins, to achieve target protein degradation by simultaneously binding two Zi^2+^ ions [44]. The data of this study show that BsIAP2 has a strong anti-apoptotic effect, especially in regulating the expression of apoptotic genes. A previous study found that deletion of the Ring domain of LyIAP2 caused the protein expression level to be increased significantly and the high molecular weight polyubiquitination signal to be inhibited by the proteasome, suggesting that LyIAP2 was self-regulated by the ubiquitination pathway, indicating that Bir and Ring of BsIAP2 are necessary for anti-apoptosis [45]. Based on this result, we speculate that BsIAP2 may be similar to IAPs in mammalian cells, which can cleave caspase and inhibit its activity [46]. In addition, BsIAP2 may regulate the expression of apoptotic genes and the activity of caspase by activating the NF-κB signaling pathway [47,48,49].

Protein domain analysis showed that BsIAP3 contains two Bir domains, and Bir1 is more conserved than Bir2 (Figure 10), indicating that Bir2 domains play a more important role in the function of BsIAP3. Similarly, BsIAP2 and BsIAP3 are similar to LyIAP, which blocked drpr-induced apoptosis in Sf9 cells to a certain extent due to the difference in the Bir domain [11,50]. Since the N-terminus of the Bir domain is responsible for protein recognition, this confers higher protein recognition specificity to BsIAP3 [51,52,53].

This study also showed that BsIAP2 and BsIAP3 had a strong inhibitory effect on the expression of the *caspase*-3 gene, but had no significant effect on the function of the Caspase-3 protein, which may be similar to the mechanism of apoptosis caused by overexpression of PKCd and PKCq [54,55]. Previous studies have confirmed that IAP protein needs to contain both Bir and Ring domains when it exerts its anti-apoptotic function. The deletion of either domain may lead to the loss of inhibitory ability, or even the opposite function [12,56,57]. In addition, the co-expression of BsIAP2 and BsIAP3 has an obvious additive effect, indicating that BsIAPs can interact at different steps of multiple apoptotic pathways such as HID, GRIM, and RPR, just like P35 protein can block multiple caspase pathways [58]. In this study, MultiBac was also used to continuously co-express multiple BsIAPs genes in Sf9 cells to explore the impact of the combination of BsIAPs on baculovirus-induced insect cell apoptosis.

## 5. Conclusions

Here, we describe the successful construction of recombinant pIE1 vector and MultiBac vectors using transient co-transfection technology and a multigene co-expression system, both of which can simultaneously overexpress multiple baculovirus BsIAP proteins. Among them, the transient expression vectors were used to explore the molecular expression effect of group II baculovirus IAP overexpression on the classical apoptosis pathway of insect cells, and the Multibac expression vectors were used to explore the effect of multiple BsIAPs’ co-expression on insect cell apoptosis induced by virus infection. The detection results of the molecular expression of the classical apoptosis pathway in insect cells and the rate of virus-induced apoptosis and progeny virus nucleocapsid production confirmed for the first time that overexpression of group II baculovirus BsIAP1 can promote apoptosis, while that of BsIAP2 and BsIAP3 significantly inhibits apoptosis. Further, combined overexpression of BsIAP2 and BsIAP3 leads to a synergistic effect, with superior antiviral-induced apoptotic effects on insect cells than those of each IAP expressed individually, to a significant degree. In addition, combined overexpression of the three BsIAPs significantly reduced the efficiency of gene expression, apoptosis, and baculovirus proliferation in insect cells.

## Figures and Tables

**Table 1:** Primers used to amplify target genes.

Name	Sequence	Size (bp)
*Bsiap1* (with His-Tag)	F: 5′-aggatccatgcatcatcaccaccatcacatgtgttgttttttttggc R: 5′-agagctcttaatttacatacaatttttg	549
*Bsiap2* (with V5-Tag)	F: 5′-aggatccatgggtaagcctatccctaaccctctcctcggtctcgattctacgatgaactacgaaagtgct R: 5′-agagctcttataaaaacactttaatc	933
*Bsiap3* (with Flag-Tag)	F: 5′-aggatccatggattacaaggatgacgacgataagatgtacatagaagatt R: 5′-agagctcttacacttgatacatgc	831
*gfp*	F: 5′-acccgggatggtgagcaagggcgagga R: 5′-agctagcttacttgtacagctcgtccat	720
*mCherry*	F: 5′-acccgggatggtgagcaagggcgagga R: 5′-agctagcttacttgtacagctcgtccatgcc	711

The 5′ ends of primers were designed to create restriction enzyme sites (underlined. *Bam*H I: ggatcc; *Sac* I: gagctc; *Xma* I: cccggg; *Nhe* I: gctagc). The shaded areas encode protein tags, for use in protein purification and western blot analysis.

**Table 2:** Primers used for Q-PCR.

Gene	Primer Sequence	Gene ID	Size (bp)
*α-Tubulin*	F: 5′-agtccagatcggtaatgc R: 5′-gctgaagaaggtgttgaag	ABX55885.1	124
*baculovirus*	F: 5′-cccgtaacggacctcgtactt R: 5′-ttatcgagatttatttgcatacaac	NC_001623.1	141
*caspase*-1	F: 5′-gattcaaagttacggtgttcccta R: 5′-ggttgtctggcttgtaatgagtat	U81510.1	171
*caspase*-2	F: 5′-gtaaggttctgattggcaattagc R: 5′-cggtacttgtggttggtgtt	KP711808.1	173
*caspase*-9	F: 5′-acacagagtttgacaacaatatcg R: 5′-ggtctcatagtccaccaacac	KC683711.1	179
*dronc*	F: 5′-ctggtagatacgcttggagaacta R: 5′-gcctgtttgatgtgctaagact	JX912275.1	160
*dredd*	F: 5′-aacaccacaaggaatggaagt R: 5′-agttacaggcatcgttggaa	KU668855.1	200
Sf9-*iap*	F: 5′-gttggagagttgtgttgtttgttt R: 5′-aatagcgttaatgttgaggaggag	AF186378.1	199
*vp39*	F: 5′-acccgataagaagcagtgac R: 5′-cccagagtagcgttaggatt	NC_001623.1	226
*p35*	F: 5′-cgaacgcaacgactactac R: 5′-tgagcaaacggcacaataac	NC_001623.1	125
*p53*	F: 5′-caccgtctcaaccgtatc R: 5′-gaggacattcttcgctattt	HM773026.1	210

## Data Availability

All datasets generated for this study are included in the article/supplementary material.

## Supplementary Materials

The following supporting information can be downloaded at: https://www.mdpi.com/article/10.3390/genes13050750/s1, Figure S1: Figure 4-Western blot-Original Image, Figure S2: Details of Flow Cytometry.
