# Supplementary material for: Impact of Group II Baculovirus IAPs on Virus-Induced Apoptosis in Insect Cells
Source: Genes (Basel). 2022 Apr 24;13(5):750. doi: 10.3390/genes13050750 (PMC9140827; doi:10.3390/genes13050750)
Supplement: Supplementary file 1 [file genes-13-00750-s001.zip › genes-1664843-non_publishedmaterial) (File S1).pdf]

Gene sequence of Group II Baculovirus *Bsiaps*

Gene sequence of *Bsiap1*

atgtgttgtttttttggcacgctgacaatataatggaatattttaatttgctaaaagacgaaaactatcgaattgatacgtttaataaaaattggccac
atggctatcctttgaccccaaaattattggccagacacggattttattataatggmgaaagcgacacaatcaaatgttgcgaatgtaaattaaca
ctcaccaacttaaaacckratcaatttattgatcgatttcatgaacgttttaaatgttcatatgcaaacgtgcgcactttcgagtattgtatacaaaac
aatttgccttatagcaacgaaaacgaaaaagatgtcgtgtccaaattagttgcagtcaaatcgtcgtcgacaattccggaaaagctcgaacaaa
acgattcacaaaattgtaacatttgcatgtcgaatgtrattaatgcctgtttggtgccatgtggtcacatgctatgtctcgagtgtgctcttgaattaa
acaacacaatttgtccttattgtagaaatgtttcaacaattcaaaaattgtatgtaaattaa-3’

Gene sequence of *Bsiap2*

atgaactacgaaagtgctataaatcgtgatttggcaccgccgtttttctacaaaaatgtgctcaaccgatttgcaacttttaacaattccatcaacct
gatggacagcgaaaagcgacaatttgcaaaacacggattttatttcgacagaacgggttatcgctgcgcgtattgtgcaacaactttaagcaaa
tttaataacaaaagctttaaatatcacacgttttctatttgcaaacggtccgttcaattgttgcgtgaaaatgaatcgttgaggcgagacagtttcaa
aaatttcaaacaagcgcgcaaaaaattcaaaggggtggcggaccggctggccgcgaacggattttattattatggcgctcgaaacgaaataa
aatgctgtgaatgtgaattggttataattaaatttagccaattcgatacactttatattgtgcataaacagtattctcctttttgcagttttacgtttggaa
cagacaatttaacaacacacgatcggtcgccttttgcacaaccgagtgcgccgccgatcgaattgataacgccgaaatcattttcggagcattc
ggatttgtctaatgagtcgaaaattataacatacaaacaagcggaatcaccagaattaaatacaccgaacgcgcacgaatcaaacgatgttaac
aatctcaaattgtatcccgtgttagcaacgacaaacgtgtctcattattttgaaagatcaaacgctttgagacctgcaaacgacgctaaaccgttgt
cagatgaaaataaaatgtgtgttgtatgttttgaaaaagaacgaactatttgtttttttgccatgtggacatgtttgcgtatgcgaattgtgcgctgaca
aatgtaaaaaaaagtgttgtttatgccgagaacttatcaaaaataagattaaagtgtttttataa-3’

Gene sequence of *Bsiap3*

atgtacatagaagattattcaaaaatgacggaagaagcaaacagactggcttcatttacaaactggcccgtggtgtttttaacgccgcagcagat
ggcaaaaaacggtttttactatattggtgtgcacgacgaagtgcgttgcgcattttgtaaagtagaatttaggaaatggatggaaggcgacaatc
cggccgatcaccatcgaaaatgggcgccacaatgtcctttttttaaataataaaatcgacgccggccaagatgtatgcggtacgcgagaagttat
ttttgccccttccccggcgcatccgcaatacgcgacaaaaacggctcggttgcgcacttttgaacgcaactggccttgcgctttgaaacaaaaa
cctgagcagttggcggatgccggttttttttacacgggccaaggcgacaagacgatttgtttcttttgcaacgggggccttaaggattgggaag
atggcgatgaaccttgggaacaacatgcgcgctggtttgataattgcatctatgttcaactagtaaaaggacgcgattacgtgcaaaatgttattt
cgaacgcttgcgttatacccgcagctaaaaagcaaatgcccaaatcggacgctacgcttgtgtcgcatgccgttgttgaggttgaaaacaagc
gcgaacttgaagattctaaagcatgccgaatttgtttcgaagaagaacgaaacgtgtgctttgtgccgtgcgggcacgtggcaacgtgtggtaa
atgcgcagtggcactacaaaactgtcctacgtgtcgtgtcaaaatcaataatgctgttcgcatgtatcaagtgtaa-3’

Gene sequence of *egfp*

atggtgagcaagggcgaggagctgttcaccggggtggtgcccatcctggtcgagctggacggcgacgtaaacggccacaagttcagcgtg
tccggcgagggcgagggcgatgccacctacggcaagctgaccctgaagttcatctgcaccaccggcaagctgcccgtgccctggcccacc
ctcgtgaccaccctgacctacggcgtgcagtgcttcagccgctaccccgaccacatgaagcagcacgacttcttcaagtccgccatgcccga
aggctacgtccaggagcgcaccatcttcttcaaggacgacggcaactacaagacccgcgccgaggtgaagttcgagggcgacaccctggt
gaaccgcatcgagctgaagggcatcgacttcaaagaggacggcaacatcctggggcacaagctggagtacaactacaacagccacaacgt
ctatatcatggccgacaagcagaagaacggcatcaaggtgaacttcaagatccgccacaacatcgaggacggcagcgtgcagctcgccga
ccactaccagcagaacacccccatcggcgacggccccgtgctgctgcccgacaaccactacctgagcacccagtccgccctgagcaaaga
ccccaacgagaagcgcgatcacatggtcctgctggagttcgtgaccgccgccgggatcactctcggcatggacgagctgtacaagtaa-3’

Gene sequence of *mCherry*

atggtgagcaagggcgaggaggataacatggccatcatcaaggagttcatgcgcttcaaggtgcacatggagggctccgtgaacggccac
gagttcgagatcgagggcgagggcgagggccgcccctacgagggcacccagaccgccaagctgaaggtgaccaagggtggccccctg
cccttcgcctgggacatcctgtcccctcagttcatgtacggctccaaggcctacgtgaagcaccccgccgacatccccgactacttgaagctgt
ccttccccgagggcttcaggtgggagcgcgtgatgaacttcgaggacggcggcgtggtgaccgtgacccaggactcctccctgcaggacg
gcgagttcatctacaaggtgaagctgcgcggcaccaacttcccctccgacggccccgtaatgcagaagaagaccatgggctgggaggcct
cctccgagcggatgtaccccgaggacggcgccctgaagggcgagatcaagcagaggctgaagctgaaggacggcggccactacgacgc
tgaggtcaagaccacctacaaggccaagaagcccgtgcagctgcccggcgcctacaacgtcaacatcaagttggacatcacctcccacaac
gaggactacaccatcgtggaacagtacgaacgcgccgagggccgccactccaccggcggcatggacgagctgtacaagtaa-3’
