# Supplementary figures and images for: Impact of Group II Baculovirus IAPs on Virus-Induced Apoptosis in Insect Cells
Source: Genes (Basel). 2022 Apr 24;13(5):750. doi: 10.3390/genes13050750 (PMC9140827; doi:10.3390/genes13050750)

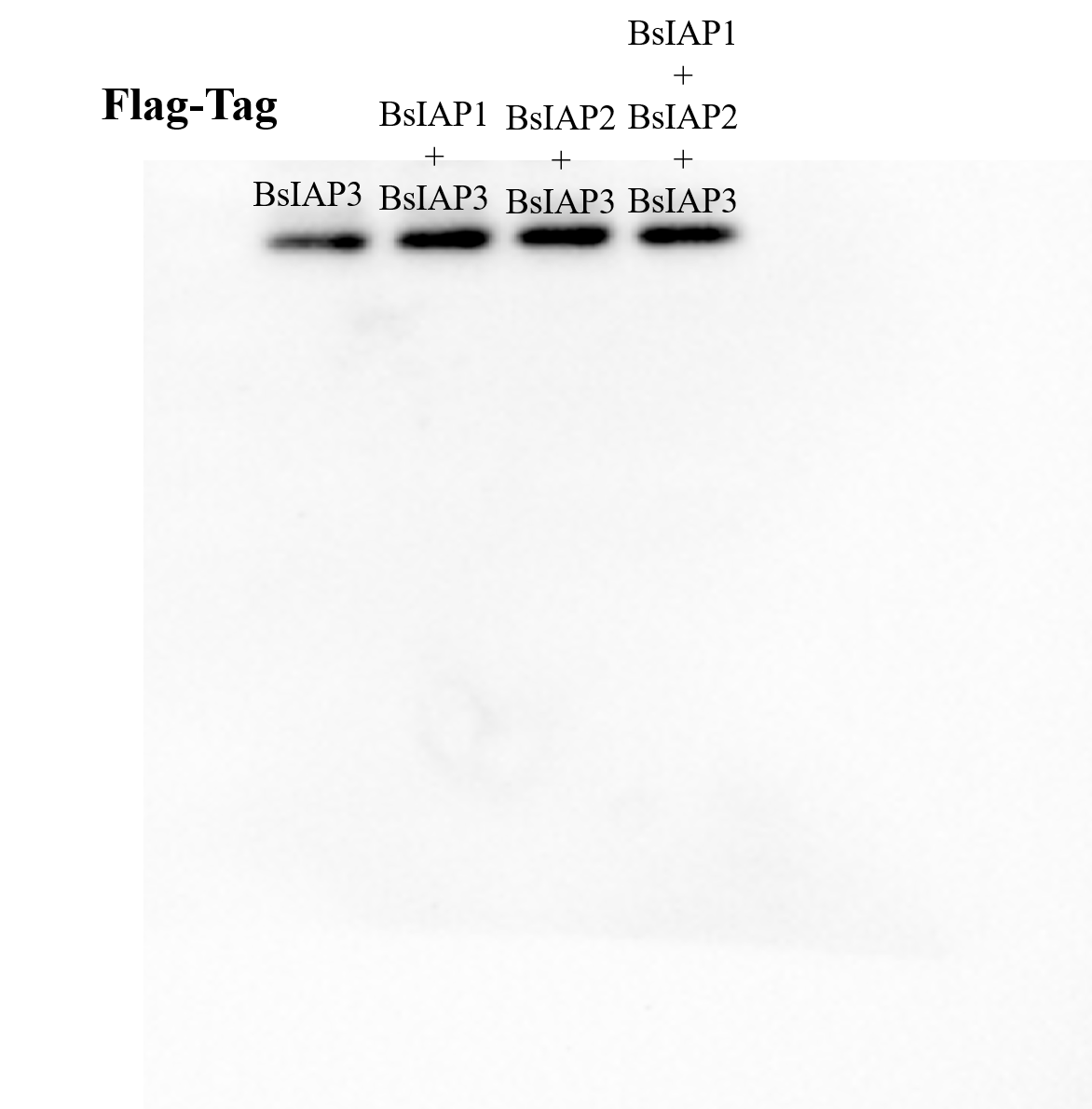

Supplement: Supplementary file 1 [file genes-13-00750-s001.zip › Figure S1ú║Figure4-Western blot-Original Image/Figure4-Western blot identification of BsIAP-expressing cell samples using Flag antibody.tif]

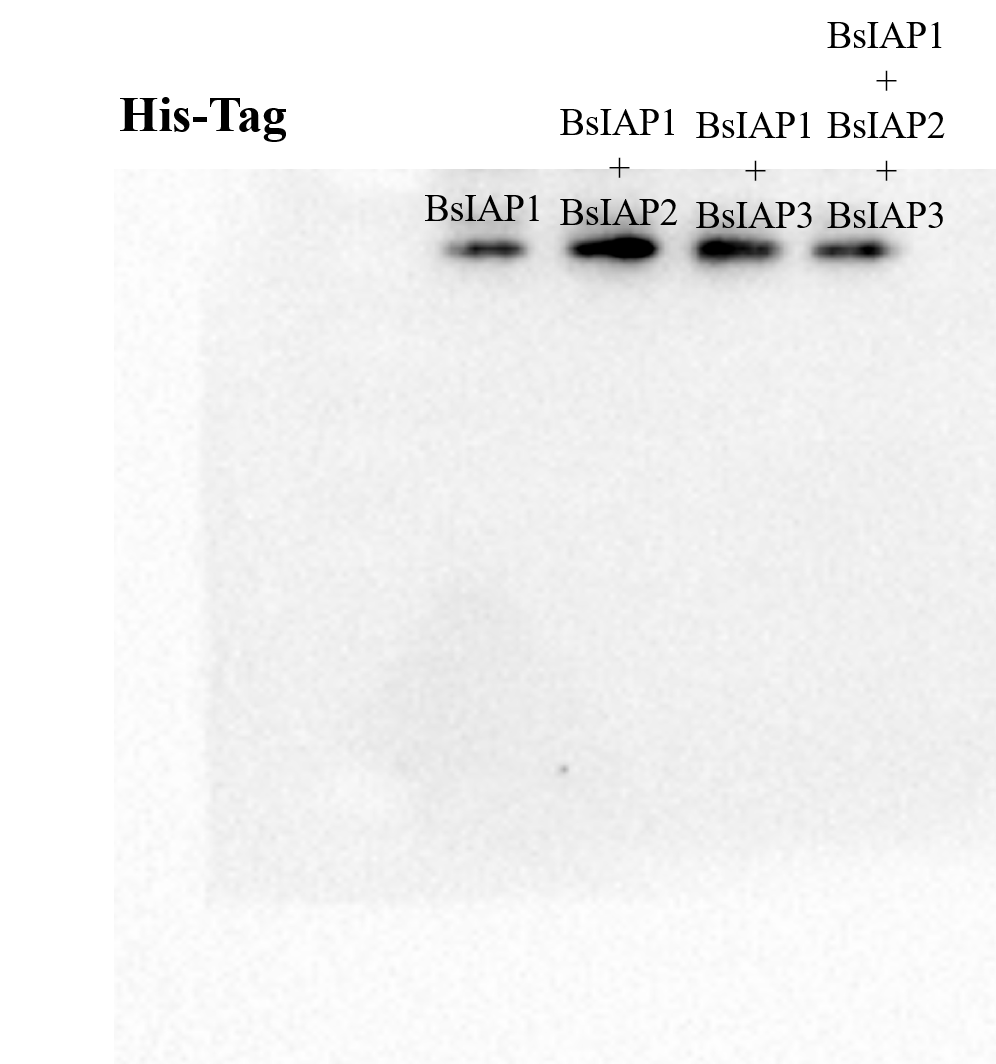

Supplement: Supplementary file 1 [file genes-13-00750-s001.zip › Figure S1ú║Figure4-Western blot-Original Image/Figure4-Western blot identification of BsIAP-expressing cell samples using His antibody.tif]

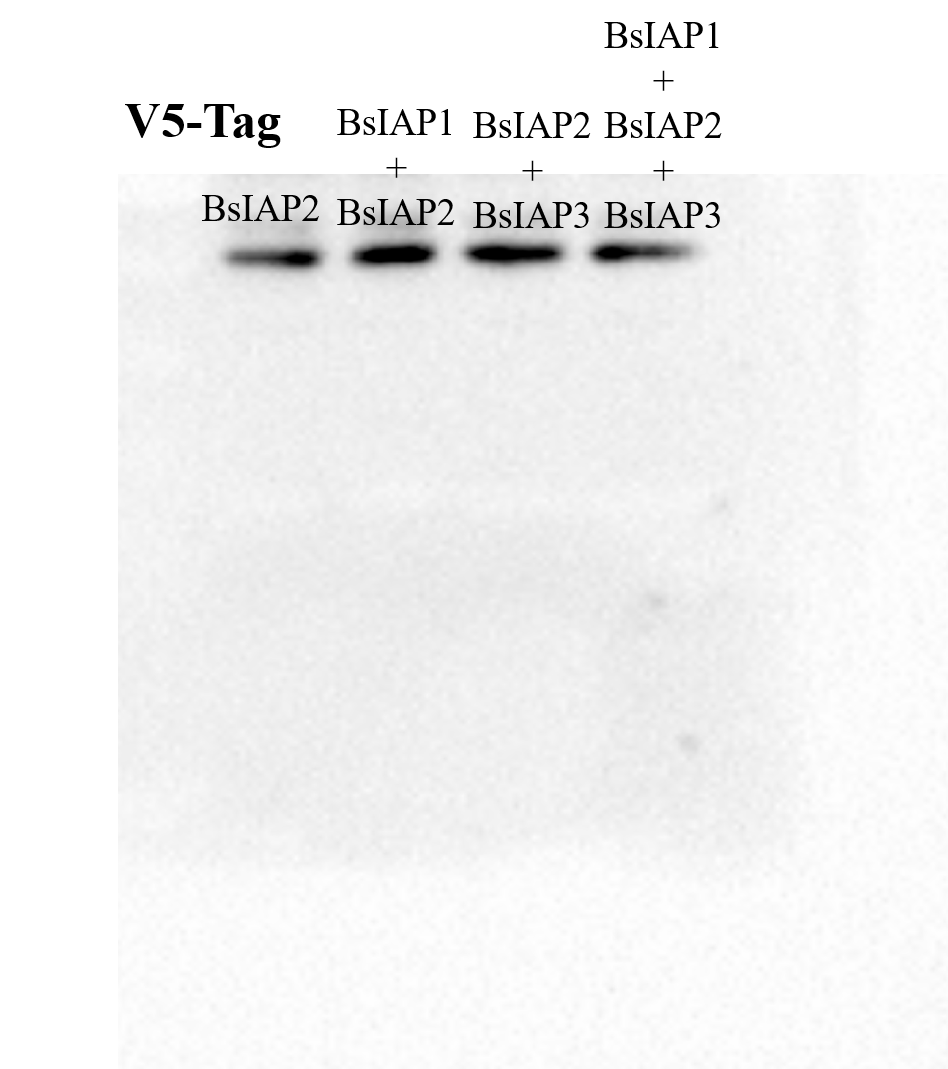

Supplement: Supplementary file 1 [file genes-13-00750-s001.zip › Figure S1ú║Figure4-Western blot-Original Image/Figure4-Western blot identification of BsIAP-expressing cell samples using V5 antibody.tif]

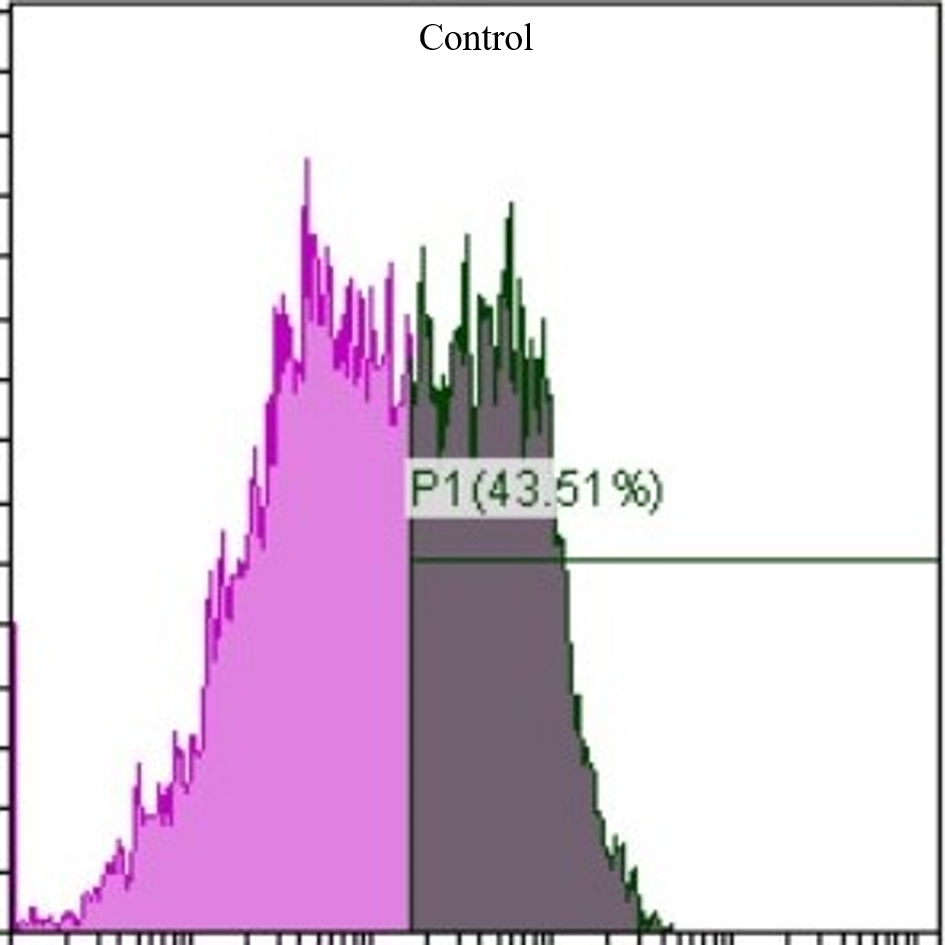

Supplement: Supplementary file 1 [file genes-13-00750-s001.zip › Figure S2ú║details of flow cytometry/Control.tif]

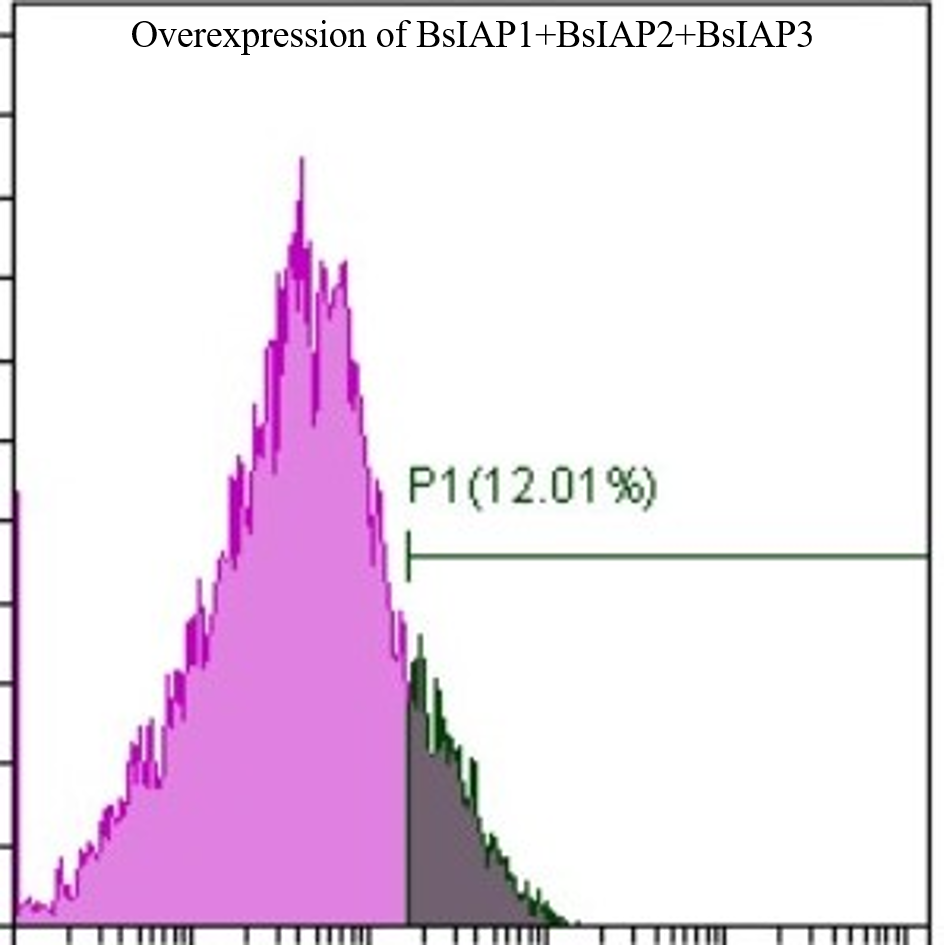

Supplement: Supplementary file 1 [file genes-13-00750-s001.zip › Figure S2ú║details of flow cytometry/Overexpression of BsIAP1+BsIAP2+BsIAP3.tif]

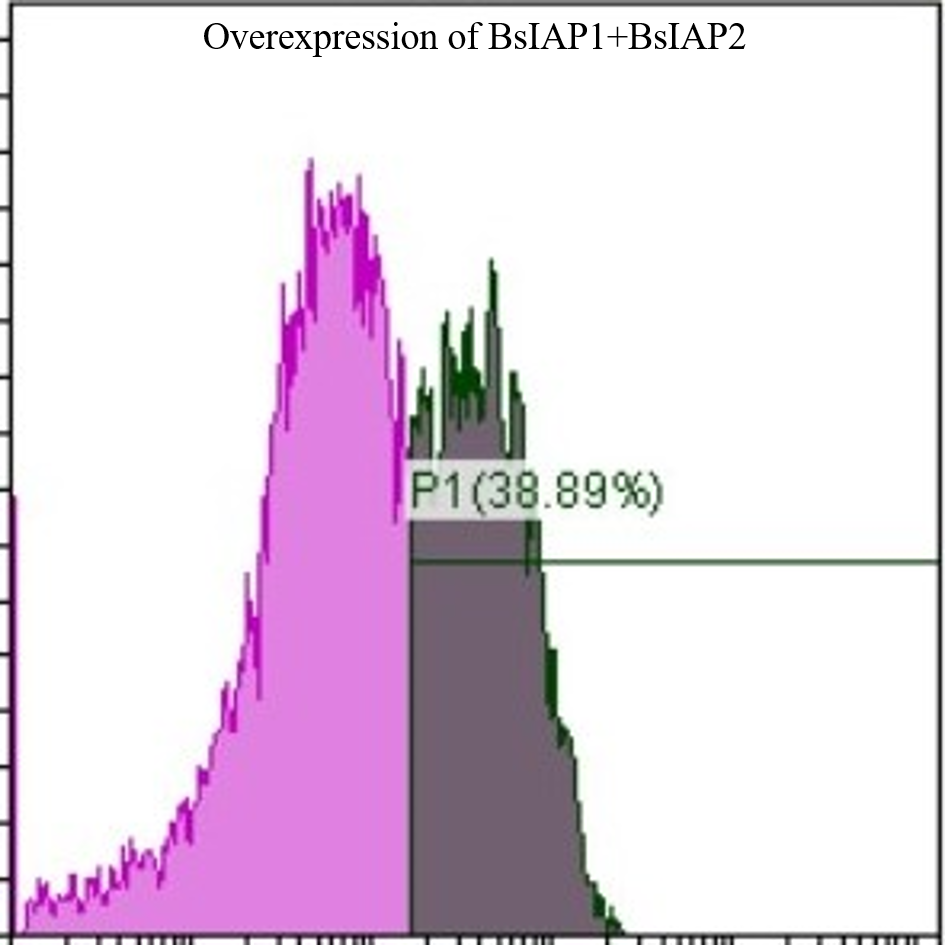

Supplement: Supplementary file 1 [file genes-13-00750-s001.zip › Figure S2ú║details of flow cytometry/Overexpression of BsIAP1+BsIAP2.tif]

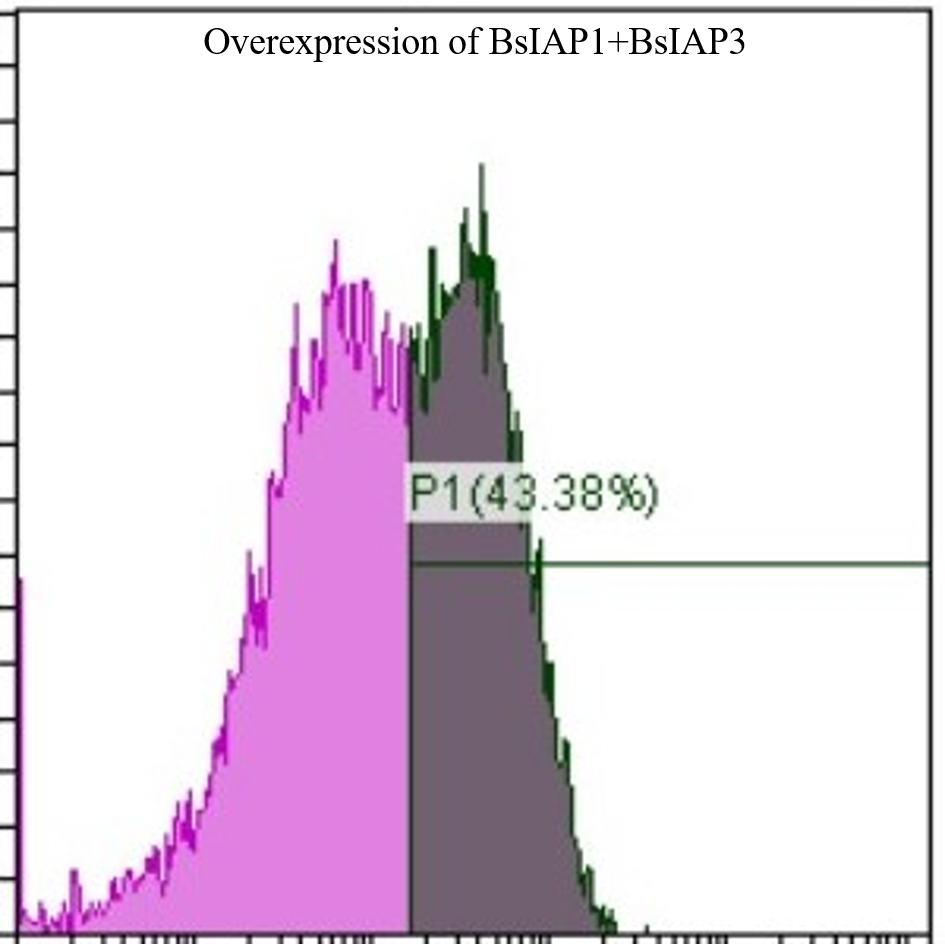

Supplement: Supplementary file 1 [file genes-13-00750-s001.zip › Figure S2ú║details of flow cytometry/Overexpression of BsIAP1+BsIAP3.tif]

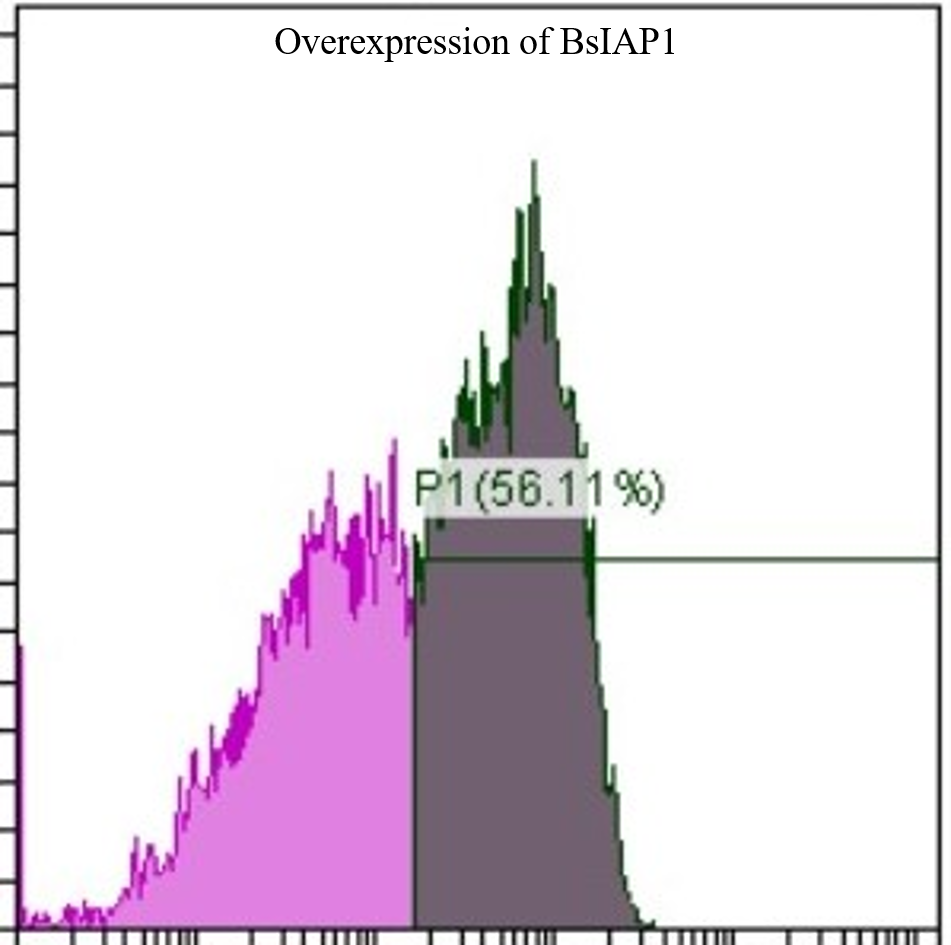

Supplement: Supplementary file 1 [file genes-13-00750-s001.zip › Figure S2ú║details of flow cytometry/Overexpression of BsIAP1.tif]

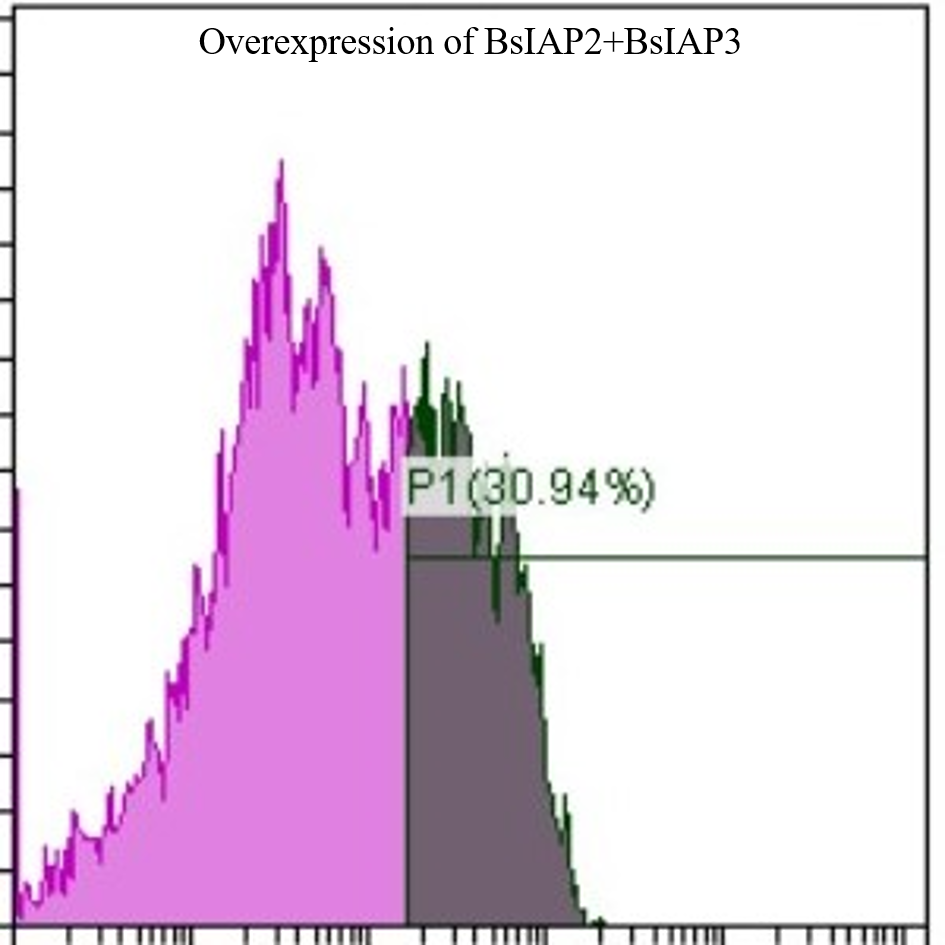

Supplement: Supplementary file 1 [file genes-13-00750-s001.zip › Figure S2ú║details of flow cytometry/Overexpression of BsIAP2+BsIAP3.tif]

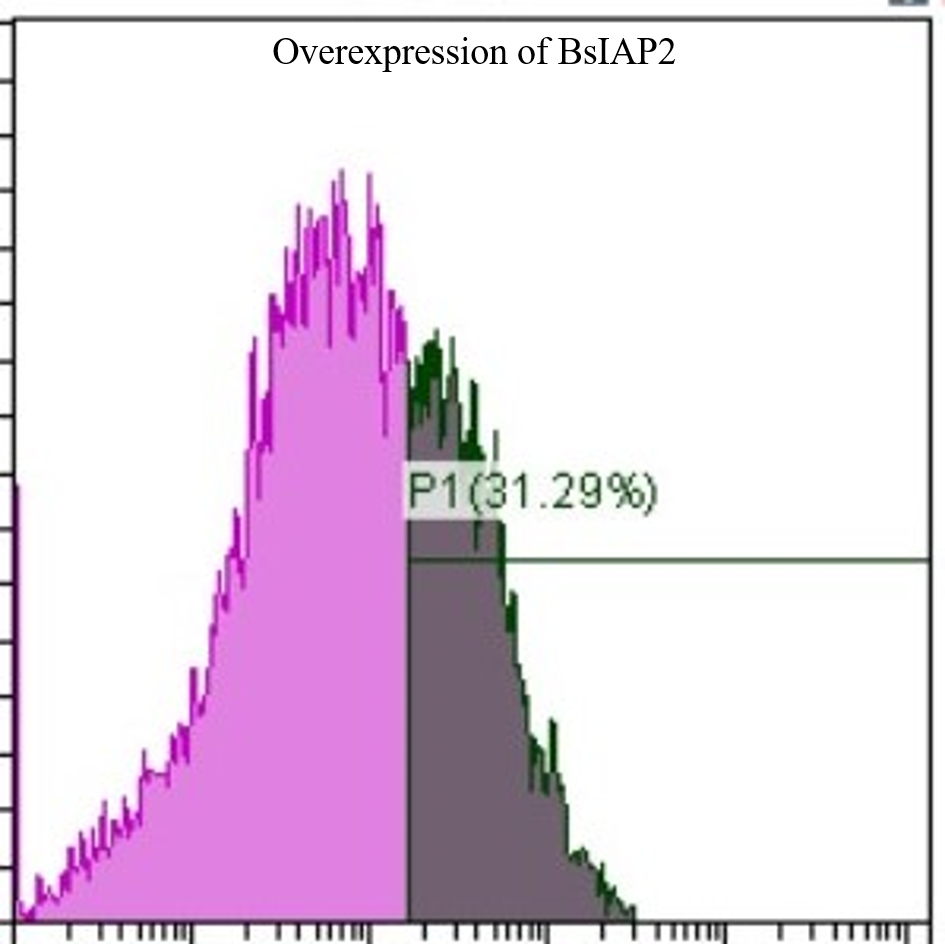

Supplement: Supplementary file 1 [file genes-13-00750-s001.zip › Figure S2ú║details of flow cytometry/Overexpression of BsIAP2.tif]

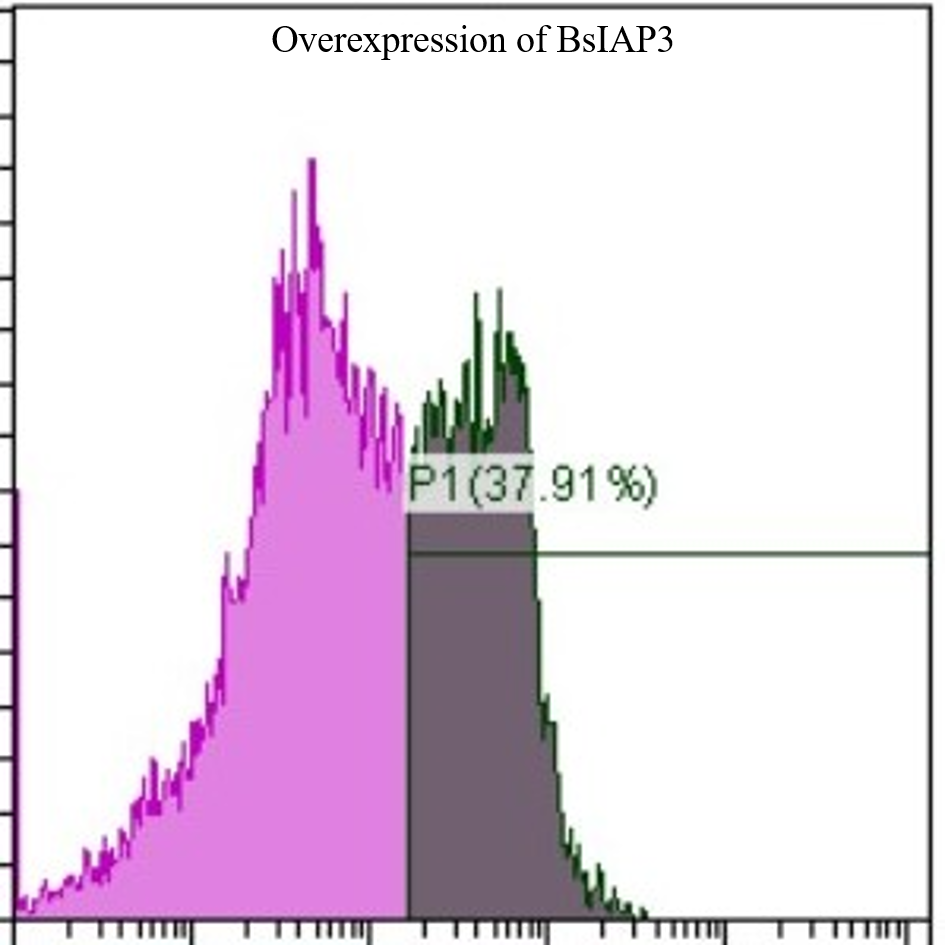

Supplement: Supplementary file 1 [file genes-13-00750-s001.zip › Figure S2ú║details of flow cytometry/Overexpression of BsIAP3.tif]
